# Improved methods for RNAseq-based alternative splicing analysis

**DOI:** 10.1038/s41598-021-89938-2

**Published:** 2021-05-24

**Authors:** Rebecca F. Halperin, Apurva Hegde, Jessica D. Lang, Elizabeth A. Raupach, C4RCD Research Group, Vinodh Narayanan, Matt Huentelman, Newell Belnap, Anne-Marie Aziz, Keri Ramsey, Christophe Legendre, Winnie S. Liang, Patricia M. LoRusso, Aleksandar Sekulic, Jeffrey A. Sosman, Jeffrey M. Trent, Sampathkumar Rangasamy, Patrick Pirrotte, Nicholas J. Schork

**Affiliations:** 1 Quantitative Medicine and Systems Biology Division, Translational Genomics Research Institute, Phoenix, AZ USA; 2 Collaborative Center for Translational Mass Spectrometry, Translational Genomics Research Institute, Phoenix, AZ USA; 3 Integrated Cancer Genomics Division, Translational Genomics Research Institute, Phoenix, AZ USA; 4 Neurogenomics Division, Translational Genomics Research Institute, Phoenix, AZ USA; 5 Yale Cancer Center, New Haven, CT USA; 6 Mayo Clinic, Scottsdale, AZ USA; 7 Northwestern Medicine, Chicago, IL USA

**Keywords:** Genome informatics, Proteome informatics, Software, Statistical methods, Melanoma, Cancer genomics, Clinical genetics, RNA splicing

## Abstract

The robust detection of disease-associated splice events from RNAseq data is challenging due to the potential confounding effect of gene expression levels and the often limited number of patients with relevant RNAseq data. Here we present a novel statistical approach to splicing outlier detection and differential splicing analysis. Our approach tests for differences in the percentages of sequence reads representing local splice events. We describe a software package called Bisbee which can predict the protein-level effect of splice alterations, a key feature lacking in many other splicing analysis resources. We leverage Bisbee’s prediction of protein level effects as a benchmark of its capabilities using matched sets of RNAseq and mass spectrometry data from normal tissues. Bisbee exhibits improved sensitivity and specificity over existing approaches and can be used to identify tissue-specific splice variants whose protein-level expression can be confirmed by mass spectrometry. We also applied Bisbee to assess evidence for a pathogenic splicing variant contributing to a rare disease and to identify tumor-specific splice isoforms associated with an oncogenic mutation. Bisbee was able to rediscover previously validated results in both of these cases and also identify common tumor-associated splice isoforms replicated in two independent melanoma datasets.

## Introduction

**Figure 1:**
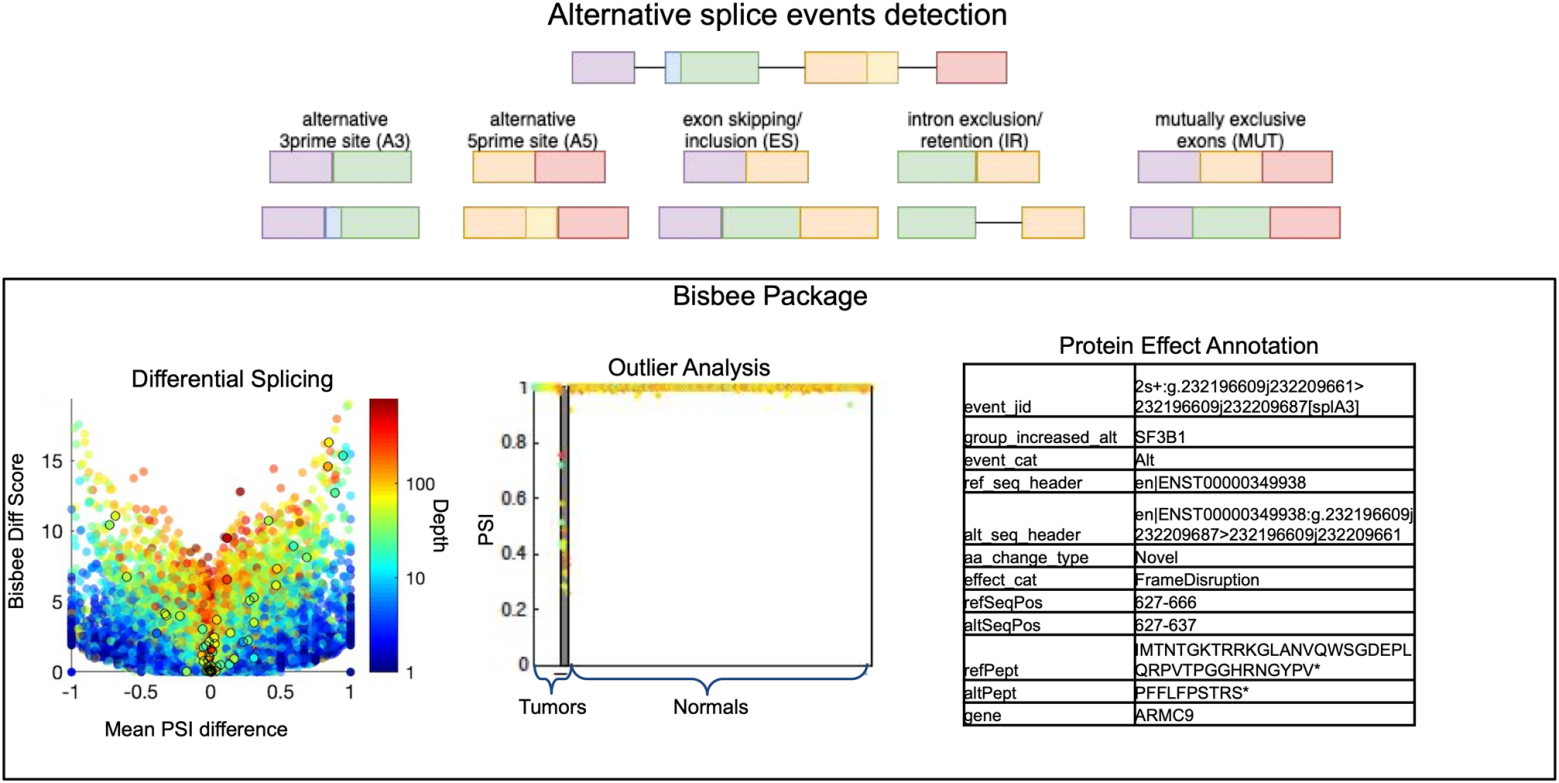
Method overview. Five types of alternative splicing events are detected by SplAdder. For each event, two alternative splice isoforms are considered. Bisbee takes the read counts supporting each isoform in each sample and performs differential splicing or outlier analysis. As illustrated in the volcano plot on the left, Bisbee Diff is able to detect high coverage events with subtle differences in percent spliced in (PSI) as well as low coverage events with large differences in PSI. As illustrated in the center plot, the Bisbee outlier test also takes into account the differences in PSI and the coverage of the event. Each dot represents a sample, with tumors on the left and normal tissues on the right. The samples are sorted by outlier score within each set on the x-axis, the PSI is plotted on the y-axis, and the color represents the depth of coverage of the event in the sample. The dots within the grey stripe pass the outlier score threshold. Bisbee also annotates protein-level effects and as can be seen in the example output on the right.

Alternative splicing has been shown to play an important role in normal cellular processes as well as a wide range of pathogenic processes underlying many different diseases^1, 2^. For example, global dysregulation of splicing, as well as mutations in genes regulating splicing, such as SF3B1, have been observed in a variety of tumors^3, 4^. In addition, the results of genome wide association studies (GWAS) focusing on common chronic conditions have identified a number of disease-associated variants that influence splicing, suggesting a role for alternative splicing in mediating many common diseases^5, 6^. Furthermore, highly penetrant variants that affect splicing have been classified as pathogenic in a number of monogenic disorders^7^. The detection of disease relevant splice alterations is not trivial, as there are hundreds of thousands of annotated splice sites in the human genome. In addition, there is also great potential for the emergence of novel unannotated splice sites at countless locations in the genome. This suggests a need for robust statistical methods for detecting and quantifying differential splice events in comparative studies in health and disease. We have developed a novel statistical framework for differential splicing and splice outlier detection. This framework identifies splice events in an individual sample based on a metric evaluating the percentage of reads supporting the event, or the ‘percent spliced in (PSI),’ which reflects evidence supporting that event beyond the range observed in a set of reference samples. The methods are implemented in a package called Bisbee designed for RNAseq data splicing analysis (Fig. 1). Bisbee also provides protein-level splicing effect predictions. We validated these predictions and benchmarked our statistical methods using normal tissue samples with both RNAseq and mass spectrometry data^8^.

Alternative splicing analysis consists of three main steps: detection, statistical comparison, and effect prediction. Here we leverage an existing tool for detection and implement new methods for the statistical analysis and effect prediction steps. Software packages for detecting splicing alterations may be broadly broken down into two categories: those that only identify events found in annotated transcripts such as ballgown^9^, MISO^10^, rMATs^11^, and SUPPA2^12^, and those that additionally detect novel splice events, such as ASPLI^13^, SplAdder^14^, SGSeq^15^, LeafCutter^16^, and MAJIQ^17^. As aberrant splicing in disease states may result in novel transcripts, we sought to identify and extend the capability of a tool that can identify novel splice events. We chose SplAdder for basic splice event detection because it has demonstrated utility in a large pan-cancer study and can enable comparisons to large sets of normal tissues from GTEx without requiring access to raw GTex data^14^. SplAdder also has advantages in terms of its modularity, facilitating analysis of large datasets in a cluster computing environment, and it also reports splice event coordinates in a straightforward manner.

Several splicing analysis packages include functions for testing differential splicing between two groups including ballgown^9^, ASPLI^13^, and SplAdder^14^. These typically use a generalized linear model and treat the overall expression level of the gene as a covariate to normalize expression differences that may confound the detection of splicing differences. However, a more straightforward approach would be to explicitly test the difference in the ‘percent spliced in’ (PSI), and therefore obviate the need to normalize for library size or expression level. We selected a beta binomial model, as they are used in many DNA sequencing variant calling strategies to model the distribution of reads supporting the existence of reference and alternate alleles^18–21^. Here we consider that each splice event has two alternate alleles, such as an exon included versus skipped in a particular gene (Fig. 1). The binomial model captures the noise in the technical measurement of the PSI value due to the depth of coverage at the splice event without needing to rely on replicates, and the beta distribution models the biological variation in the splicing. Our proposed approach is most similar to the strategy implemented in the ‘LeafCutter’ program which uses a multinomial to test for differences in intron usage within a region, reporting clusters of likely splicing disruption rather than defined event types such as exon skipping^16^. We chose to work with defined splice event types for improved interpretability and potential for insight into the mechanism of splice dysregulation.

The detection of splice events that are specific to an individual when compared to a large reference set of samples is useful in several clinical applications. This outlier analysis may be used to identify disruption of splicing due to somatic mutations or expression of known tumor-specific splice isoforms in an individual’s tumor^22, 23^. The analysis of splicing outliers may also be used to identify splice variant-induced antigens in a target individual’s tumor that do not exist in normal tissues^3, 23–25^. In addition, some rare Mendelian disorders are caused by variants that disrupt splicing. These disruptions may be detected by comparing an individual transcriptome to a set of reference samples. We are aware of only one tool designed for detecting splicing outliers in individual genomes, LeafCutterMD^26^, which does not enable the prediction of the effect of the splice variant on an encoded protein.

Predicting the protein level impact of a splice variant is critical for understanding the biological implications and potential mechanisms underlying disease states, yet most RNAseq alternative splicing analysis packages do not incorporate an effect prediction component. Splice variants may result in truncations or deletions at the protein level that result in a loss of protein function. Alternatively, spliced protein isoforms may also exhibit qualitative differences in function. For example, BCL2L1 splice isoforms have opposing effects on apoptosis^27^. In addition to identifying the expression of such known isoforms, it would also be possible to predict the functional consequences of novel splice isoforms using the domains impacted and other in silico approaches. Alternative splicing may also give rise to novel protein sequences in a cancer cell that could be recognized by the immune system^3, 23–25^.

Genomics, transcriptomics, and proteomics are being used together more often in an effort to better characterize phenotypic effects resulting from genomic alterations and pathway dysregulation^28–30^. There are many existing proteogenomics pipelines that use transcriptome sequencing to generate protein sequence databases for matching mass spectra^31–35^. Many of these pipelines take a comprehensive approach using all detected splice junctions and translating them in all six reading frames^31–36^. Such studies have been extremely useful for elucidating gene structure and cataloging splice junctions in specific samples^31, 37^. Other proteogenomics pipelines leverage transcript assembly and generate protein sequences via either three frame translation or translation of open reading frames of the reconstructed sequences^38–40^. However, transcriptome assembly is computationally-intensive and not necessary for integration with mass spectrometry, as only peptides rather than full length proteins, are detected. We are aware of only one other proteogenomics pipeline that predicts protein sequences from splice events rather than junctions or assembled transcripts; however, it does not detect events involving novel splice sites^41^. Our study utilizes splice event-level analysis as it is more amenable to comparisons between samples and facilitates interpretation.

In order to benchmark Bisbee’s methods against similar approaches we developed a ‘truth set’ with splice events validated through the detection of corresponding protein isoforms. This truth set was generated using mass spectrometry and RNAseq data on a set of normal tissues from Wang et al.^8^. We identified several other splice variant analysis tools to consider for benchmarking against Bisbee. However, only a few of them provide utilities for predicting the effect of splice alterations at the protein level, which would be necessary for use with our mass spectrometry truth set. Using real data with complementary measurements provides a more robust framework for benchmarking and validation. Our truth set takes advantage of the naturally occurring differences in splicing between different tissues^30^ to evaluate Bisbee’s differential splicing and splice outlier modules.

## Results

### Predicted splice isoforms are detected at the protein level

**Figure 2:**
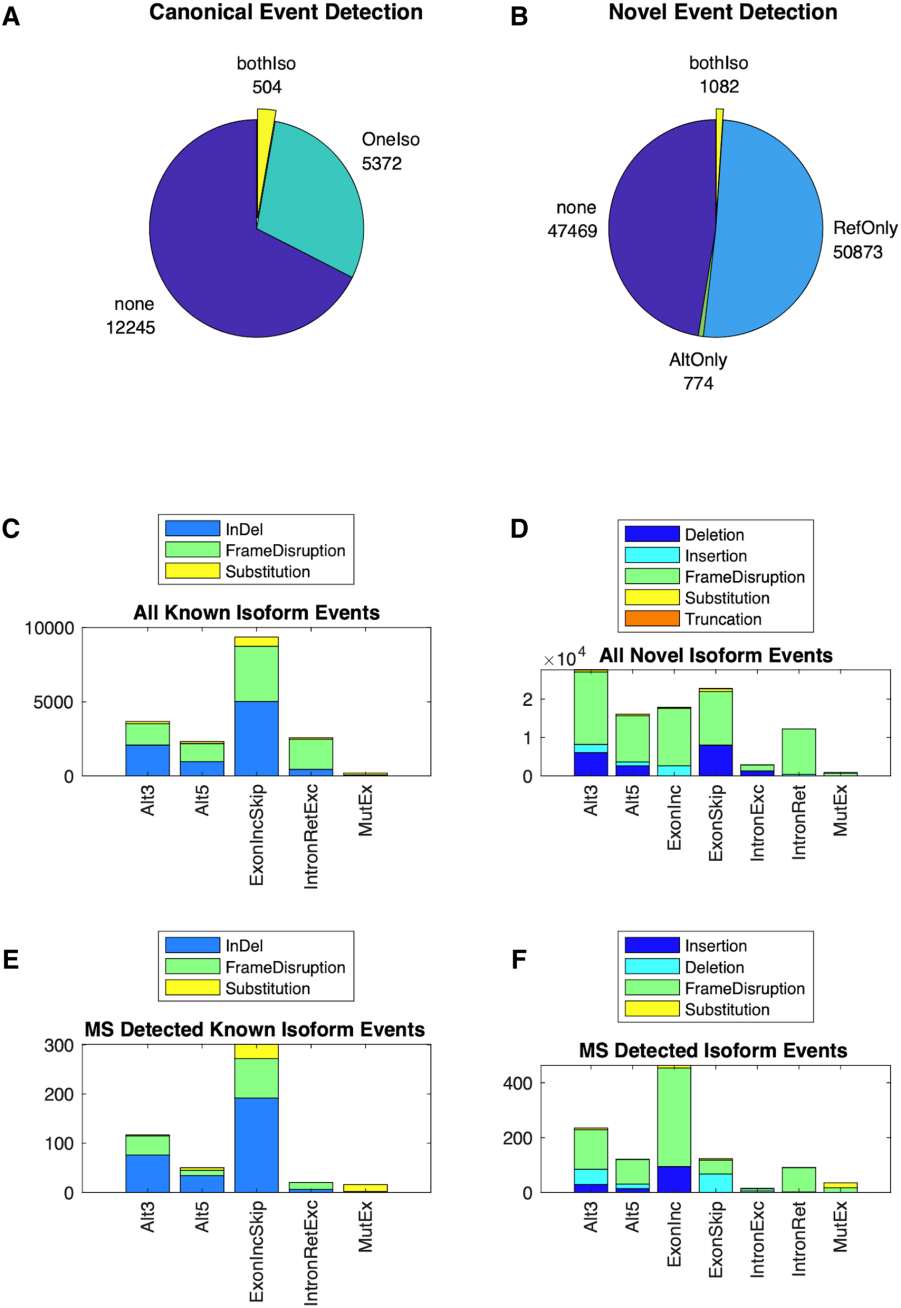
Splice event detection. Pie charts show the breakdown of splice events by their mass spectrometry evidence with “none” indicating no peptides map to either isoform, “oneIso”, indicating at least one peptide maps to one of the two isoforms of a known event, “refOnly” indicating at least one peptide maps to the reference isoform of a novel event, “AltOnly” indicating at least one peptide maps to the alternative (novel) isoform of a novel event, and “bothIso” indicating at least one peptide maps to each of the two isoforms for known (**A**) or novel (**B**) events. Breakdown of splice events by event type and predicted protein level effect for all predicted known isoform events (**C**), all predicted novel isoform events (**D**), known isoform events with both isoforms detected (**E**), and novel isoform events with both isoforms detected (**F**).

In order to validate the existence of proteins/peptides corresponding to splice variants, we leveraged a dataset from Wang et al*.*, which includes paired RNA-seq and proteomics data from normal tissues^8^. In this validation dataset, SplAdder identified 268,791 total splice events, of which 125,683 were predicted by Bisbee to be protein coding. The mass spectrometry searches identified 182,662 unique peptides (Supplemental Fig. 1). Protein evidence of alternative splicing, defined as having at least one peptide supporting each of the two isoforms, was detected for 1587 of the protein-generating events, including 1082 generating novel sequences (Fig. 2). The event categories that generate longer stretches of altered sequence have higher proportions of protein level detection as expected (Supplemental Fig. 2). We observed 330 events showing tissue specific detection patterns at the protein level, and these were used for benchmarking and validation.

### Bisbee Diff more accurately detects differentially spliced isoforms

The beta binomial differential splicing test implemented in Bisbee has one parameter that requires tuning, ${\omega }_{M}$, which is a constraint on the parameter $\omega$ which controls the overall shape of the beta distribution, particularly in its tails. In order to avoid overfitting, we reserved the tissue-specific protein isoforms dataset from Wang et al. to compare the accuracy of differential splicing methods^1^. GTEx was used for parameter optimization and threshold selection. We compared the distribution of the test statistic for the Bisbee Diff test between sets of samples from the same tissue versus different tissues using different values of the ${\omega }_{M}$ parameter. The percentage of events passing a given threshold in the ‘different’ versus the ‘same’ comparison is used as an indicator of the specificity of the test, while the percentage of events in the different comparisons passing the thresholds is used as an indicator of sensitivity. Setting the ${\omega }_{M}$ parameter to 200, and using a log likelihood ratio (LR) threshold of 8 provides optimal enrichment of splice events detected as different between different tissues compared to splice events detected as different between samples from the same tissue (Supplemental Fig. 2a).

**Figure 3:**
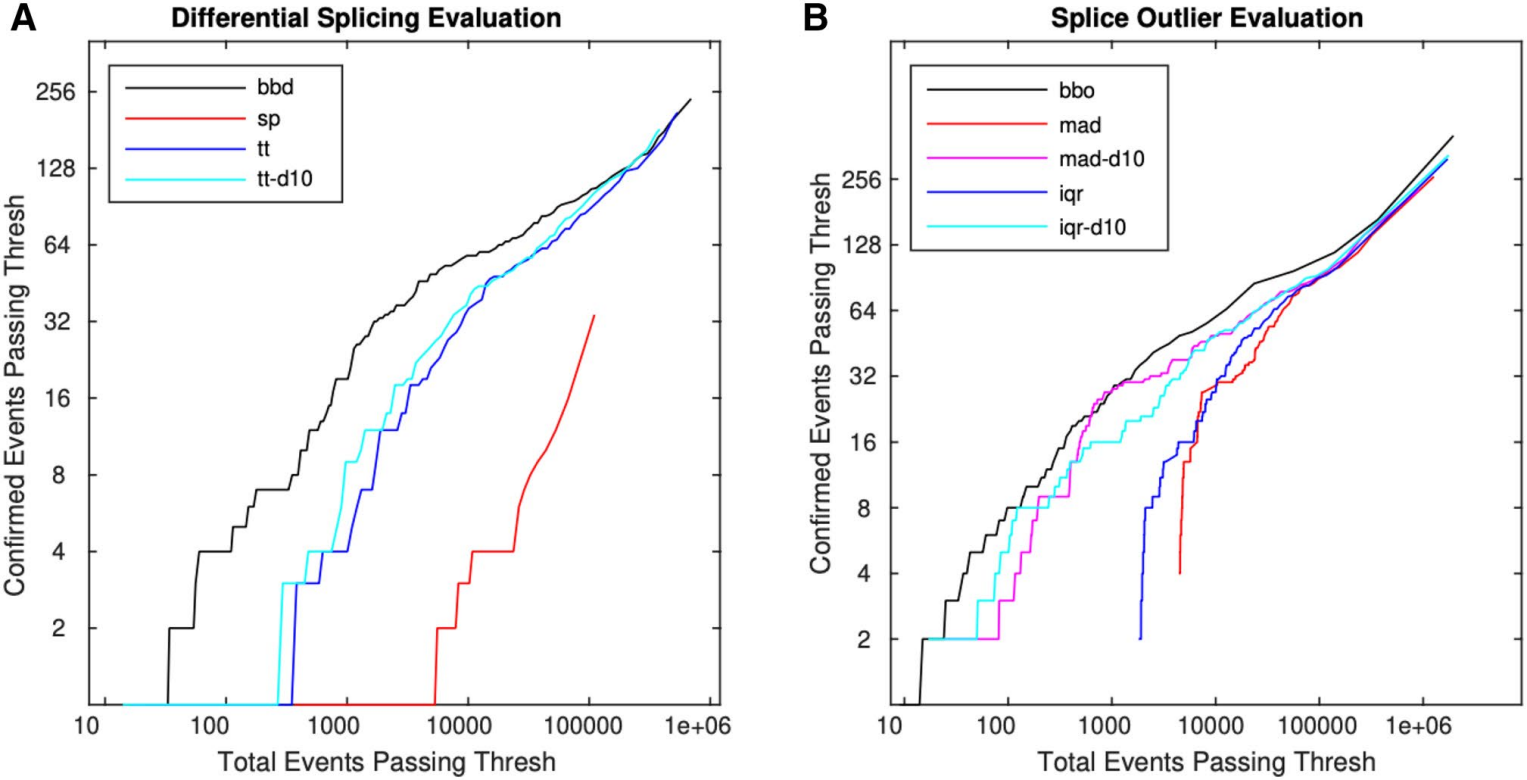
Bisbee detects protein expression confirmed splice events with high sensitivity. (**A**) The number of events with protein expression evidence of differential splicing is plotted against the total number of events passing the threshold for four different differential splicing methods: beta binomial (bbd—black), SplAdder’s test (sp—red), t-test on all PSI (tt—blue), t-test on PSI with depth > 10 (tt-d10—cyan). (**b**) The number of mass spectrometry confirmed outlier events is plotted against the total number of events passing the threshold for five different methods: bisbee outlier (black), median absolute deviation (red), median absolute deviation with depth > 10 (magenta), interquartile range (blue), and interquartile range with depth > 10 (cyan).

**Figure 4:**
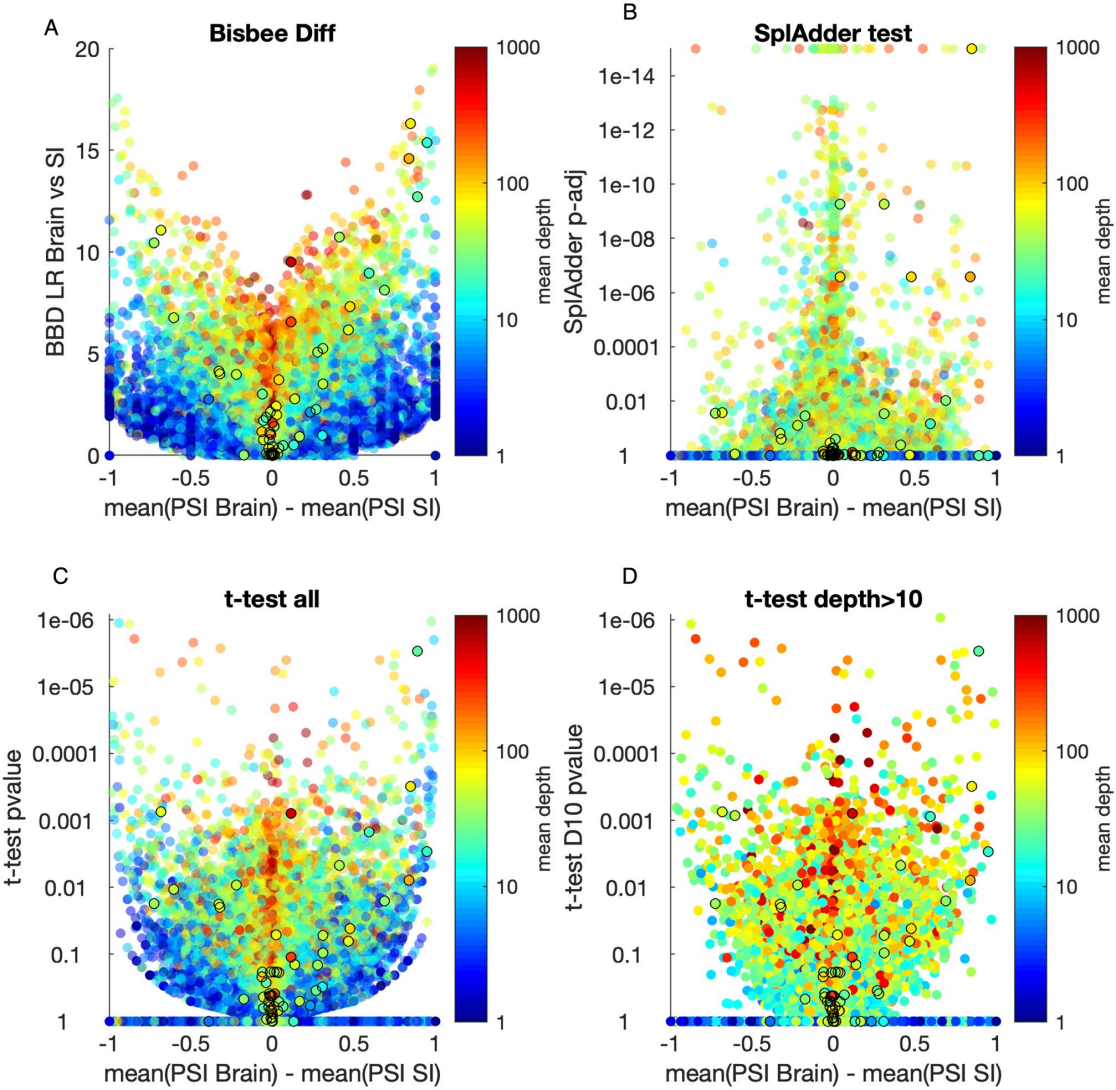
Volcano plots of brain versus small intestine for four differential splicing methods. Differential splicing results for the brain versus small intestine comparison from the Wang et al. dataset. The Bisbee Diff LR (**A**), SplAdder test adjusted *p* value on a log scale (**B**), or t-test *p* value on a log scale (**C**,**D**) are plotted against the difference in mean PSI between the brain and small intestine samples. Points are colored by the mean read depth covering the event on a log scale as indicated by the color bar. Events with mass spectrometry confirmed tissue specific protein expression are outlined by black circles.

We identified 281 instances of protein expression-confirmed isoform switches over six pairwise tissue comparisons, which represent 196 unique isoform switch events. For comparison, SplAdder’s test module was run as an example of a program that uses a, generalized linear model approach. As a simple approach, a t-test on the PSI values was pursued both using all of the PSI values regardless of depth and only including PSI values with a sequencing read depth at the position of greater than 10. To evaluate these methods, we compared the total number of events passing a given threshold to the number of protein confirmed events passing the threshold. The Bisbee Diff method consistently found higher enrichment of confirmed events out of total events passing a threshold (Fig. 3A, Supplemental Table 1). In order to see how the magnitude of PSI differences and the read depths of the events influence the performance of each of the differential splicing tests we made a volcano plot of the brain versus small intestine comparison (Fig. 4).

### Bisbee outlier more accurately detects splice outliers

The Bisbee outlier detection method parameter ${\beta }_{M}$ was optimized using GTEx data. The percentage of outlier scores passing a threshold for models trained on the same tissue was compared to the percentage passing for matching tissue models. We found a ${\beta }_{M}$ value of 80 provides the best enrichment of different tissue outliers with a log likelihood (LL) cutoff of 10 (Supplemental Fig. 2b). We used these values for benchmarking on the Wang et al. dataset with matching proteomics data^8^. We used a set of GI tissues as the reference set and detected outliers in three other tissues. We identified 140 outlier events across the three tissues, which represents 134 unique outlier events. Since we are not aware of another tool that is able to detect splice outliers and generate predicted protein sequences, we implemented two simple methods using the distribution of PSI values in the reference dataset. The first simple outlier method uses the median absolute deviation (mad) and the second using the interquartile range (iqr) of the PSI values. For both of these methods we performed the analysis both with using PSI values for all data points as well as using only PSI values for data points with a depth greater than 10. The Bisbee outlier method detected more proteomics-confirmed events for similar numbers of total events passing the same score threshold (Fig. 3B, Supplemental Table 2).

### Case study: detection of a splice event in rare disease

In order to examine the utility of the Bisbee package for research and clinical applications, we analyzed disease-causing splice mutation in the nuclear-encoded mitochondrial methionyl-tRNA formyltransferase (MTFMT)^42–44^. We previously identified homozygous mutation (c. 626 C > T) in the MTFMT gene in three children from two unrelated families (Clinvar Accession#VCV000039827.4) with Leigh syndrome and combined oxidative phosphorylation (OXPHOS) deficiency. The MTFMT mutation c. 626 C > T in the coding region resulted in a Ser209Leu (S209L) amino acid substitution, which is likely a non-pathogenic event. However, c.626 C > T is predicted to generate a splicing suppressor that results in skipping of exon 4, leading to frame shift and truncation of the protein (p. R181SfsX5)^42, 43, 45^. The c.626C site 20 base pairs (bp) upstream of the 3′ end of exon 4 is predicted to eliminate the two overlapping exonic splicing enhancers (ESE) (GT**C**AAG and T**C**AAGA) and generate an exonic splicing suppressor (ESS) sequence (GTTGT**T**)^46, 47^. To confirm the expected exon skipping and truncation, we performed differential splicing analysis of RNA sequencing data obtained from the primary fibroblast cells from three patients carrying the homozygous c. 626 C > T mutation and five unaffected controls using Bisbee. We found that the MTFMT exon 4 skipping event was the 14th highest scoring differentially spliced event. Though the LR (7.999) was just barely below the optimal threshold determined in the GTEx analysis, its high rank makes it likely to be considered in a candidate variant analysis. It is not surprising that the event did not quite pass the threshold as coverage of the event in the cases was only 10, 6 and 2 reads. If we use the protein effects predictions to filter down to events predicted to generate novel sequences that were expressed more highly in the cases compared to the controls, we find that the MTFMT exon 4 skip is the highest scoring of these events (Supplemental Fig. 3).

When trying to discover the causal variant in a rare disease, there is often only one affected case available for sequencing, so we also ran Bisbee outlier analyses on each of the three cases to illustrate the single case scenario. Since it is desirable to have a large set of reference samples for outlier analysis, but there are technical differences in the sequencing between GTEx and this dataset, we performed the outlier analysis both using GTEx fibroblasts as the reference samples and using the five unaffected fibroblast samples used in the differential splicing analysis as the reference samples, and used the minimum score of the two analysis. The Bisbee outlier scores for the MTFMT exon 4 skip in the cases were 4.8, 10.6, and 3.4, ranking 386, 16, and 1746 of all events (Supplemental Fig. 3). When only considering events generating novel protein sequences with increased expression in the cases, the MTFMT event ranked 145, 2, and 587, respectively, in each of the three cases. Despite the very low coverage of the event in the cases, Bisbee was still able to rank the event in the top 1% of all events in all three cases.

The Bisbee annotation output is shown for the MFTMT exon 4 skipping event in Table 1. Each event is assigned a unique identifier (event_jid) using the contig, strand, and junction coordinates to facilitate comparing results between datasets. The effects at the transcript (event_cat) and protein level (effect_cat) are described, as well as whether the splice event is found in ensembl transcripts (aa_change_type). The sequence headers of the two isoforms are provided in order to locate the protein sequences in the fasta output. The sample group with increased expression of the isoform labeled “alt” is indicated (group_increased_alt). The location with the protein sequence as well as the altered amino acid sequence fragments are also provided. These results confirm the expected R181SfsX5 frame shift truncation.

**Table 1:** Example Bisbee output.

event_jid	15s-:g.65312610j65313852_65313954j65316010j65312610j65316010[sp|ES]
event_cat	ExonSkip
effect_cat	FrameDisruption
aa_change_type	Novel
group_increased_alt	CASE
ref_seq_header	en|ENST00000220058
alt_seq_header	en|ENST00000220058:g.65.65312610j65313852_65313954j65316010 > 65312610j65316010|ENST00000220058:g.65312610j65319169 > 65312610j65316010_65316132j65319169
refSeqPos	181–390
altSeqPos	181–186
refPept	RFDVGPILKQETVPVPPKSTAKELEAVLSRLGANMLISVLKNLPESLSNGRQQPMEGATYAPKISAGTSCIKWEEQTSEQIFRLYRAIGNIIPLQTLWMANTIKLLDLVEVNSSVLADPKLTGQALIPGSVIYHKQSQILLVYCKDGWIGVRSVMLKKSLTATDFYNGYLHPWYQKNSQAQPSQCRFQTLRLPTKKKQKKTVAMQQCIE*
altPept	SSFQF*

### Application to TCGA Uveal Melanoma dataset

**Figure 5:**
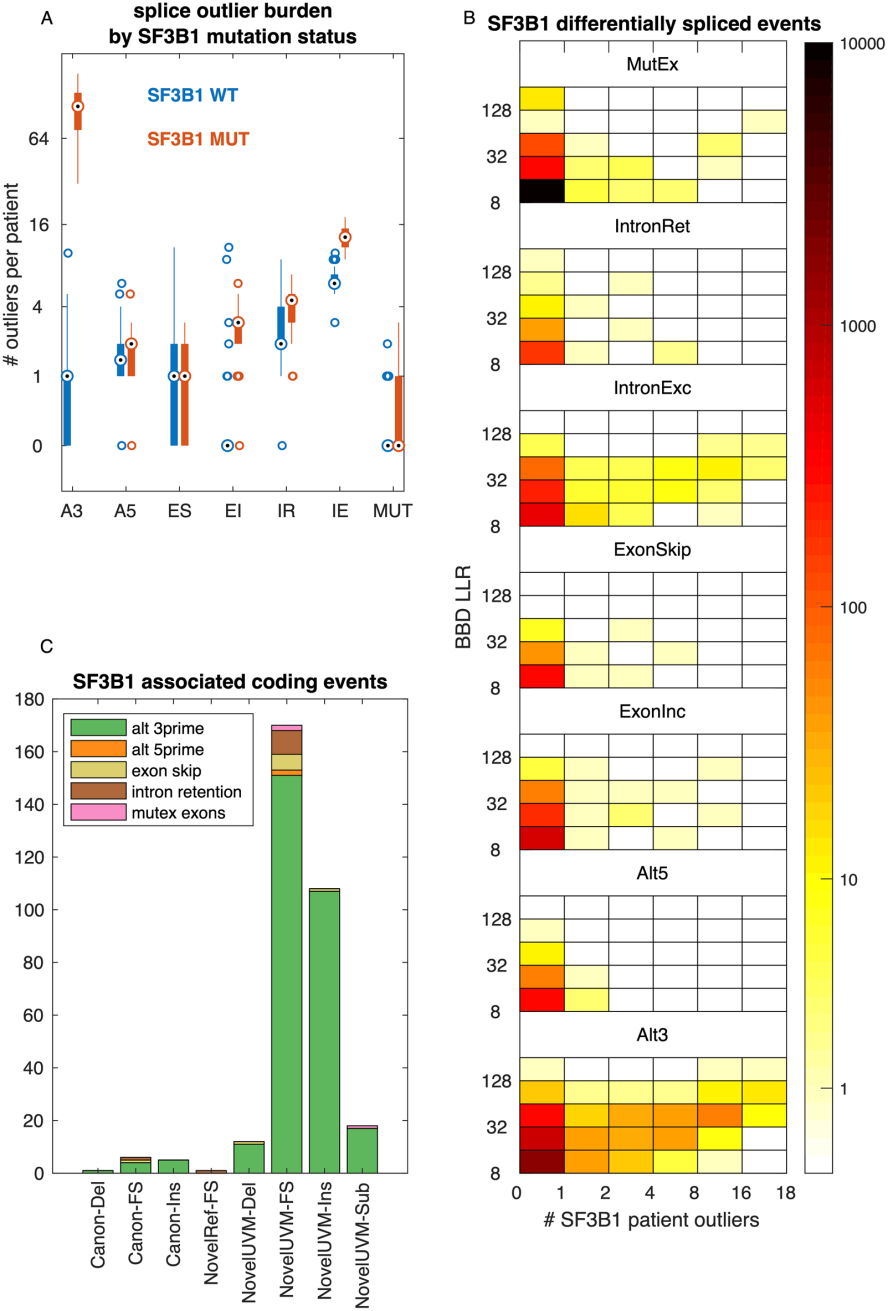
Aberrant splicing in uveal melanoma. (**A**) Boxplot comparing the number of splice event outliers between SF3B1 mutant patients (n = 18) and wild-type patients (n = 62) by splice event type. (**B**) Heatmap overview of differentially spliced events between SF3B1 mutant and wild-type tumors. Bins on the x-axis indicate the number of SF3B1 mutant tumors meeting the outlier criteria and bins on the y-axis indicate the Bisbee Diff LLR, and the color indicates the number of events falling into each bin. (**C**) Event type and protein level effects of events that are differentially spliced between SF3B1 mutant and wildtype tumors, are outliers in at least one SF3B1 mutant tumor, and result in an altered protein sequence.

We selected the TCGA uveal melanoma dataset as an example application as there is a recurrent mutation in the splicing factor 3B1 gene (SF3B1) that has been previously shown to cause aberrant 3′ splice site usage^48, 49^. To identify tumor-specific splice events, we performed Bisbee Outlier analysis using the complete GTEx tissue library exempt of testis tissue samples. Testis was excluded as it may express developmentally restricted proteins not found in normal somatic tissues^39, 40^. We also used the TCGA normal samples as a reference and took the minimum score of the two analyses. In examining the total number of splice outliers per patient, we observed a large increase in alternative 3′ splice site outliers with SF3B1 mutation as well as significantly increased exon skipping, intron retention, and mutually exclusive exon outlier burden (Fig. 5A, rank sum *p* value < 0.01). We also ran Bisbee Diff to identify differentially spliced events between SF3B1 mutant and wild-type tumors. We found 19,950 differentially spliced events of which 72% were mutually exclusive exons and 15% were alternative 3′ splice sites. The alternative 3′ differentially spliced events had higher Bisbee Diff LR and a greater overlap with events also observed in the outlier analysis (Fig. 5B). Alsafadi et al*.* previously identified differentially spliced events between SF3B1 mutant and wild-type tumors in an independent dataset, and selected seven of these events to validate in isogenic cell lines using a mini-gene splice assay^49^. All seven of these events were detected as differentially spliced by Bisbee Diff (Table 2).

**Table 2:** Validated SF3B1 mutant versus wild-type differentially spliced events.

event jid	gene	effect cat	BBD LLR	BBD rank	fit PSI SF3B1	fit PSI wt	# SF3B1 mutant tumor outliers
6s+:g.10723474j10724789 > 10723474j10724803[splA3]	TMEM14C	Silent	148.9	16	0.76	0.03	18
1s−:g.101458310j101460666 > 101458296j101460666[splA3]	DPH5	Frame disruption	87.5	33	0.78	0.00	18
2s+:g.232196609j232209661 > 232196609j232209687[splA3]	ARMC9	Frame disruption	80.2	41	0.66	0.00	18
14s+:g.75356052j75356581 > 75356052j75356600[splA3]	DLST	Frame disruption	76.6	44	0.50	0.01	0
18s−:g.683380j685921 > 683395j685921[splA3]	ENOSF1	Insertion	74.2	47	0.71	0.03	16
6s+:g.35255622j35258030 > 35255622j35258043[splA3]	ZNF76	Frame disruption	53.8	194	0.44	0.03	11
16s+:g.843274j844034 > 843274j844054[splA3]	CHTF18	Frame disruption	25.1	1316	0.30	0.00	8

In order to identify protein isoforms that may be specific to SF3B1 mutant tumors, we selected splice events that were common between the differential splicing and outlier analysis (494) and then identified those predicted to result in altered protein sequence (321). These events are primarily alternative 3′ events causing insertions or frame disruptions resulting in novel protein isoforms in the uveal melanoma tumors (Fig. 5C).

### Replication of common melanoma associated splice events in an independent dataset

**Figure 6:**
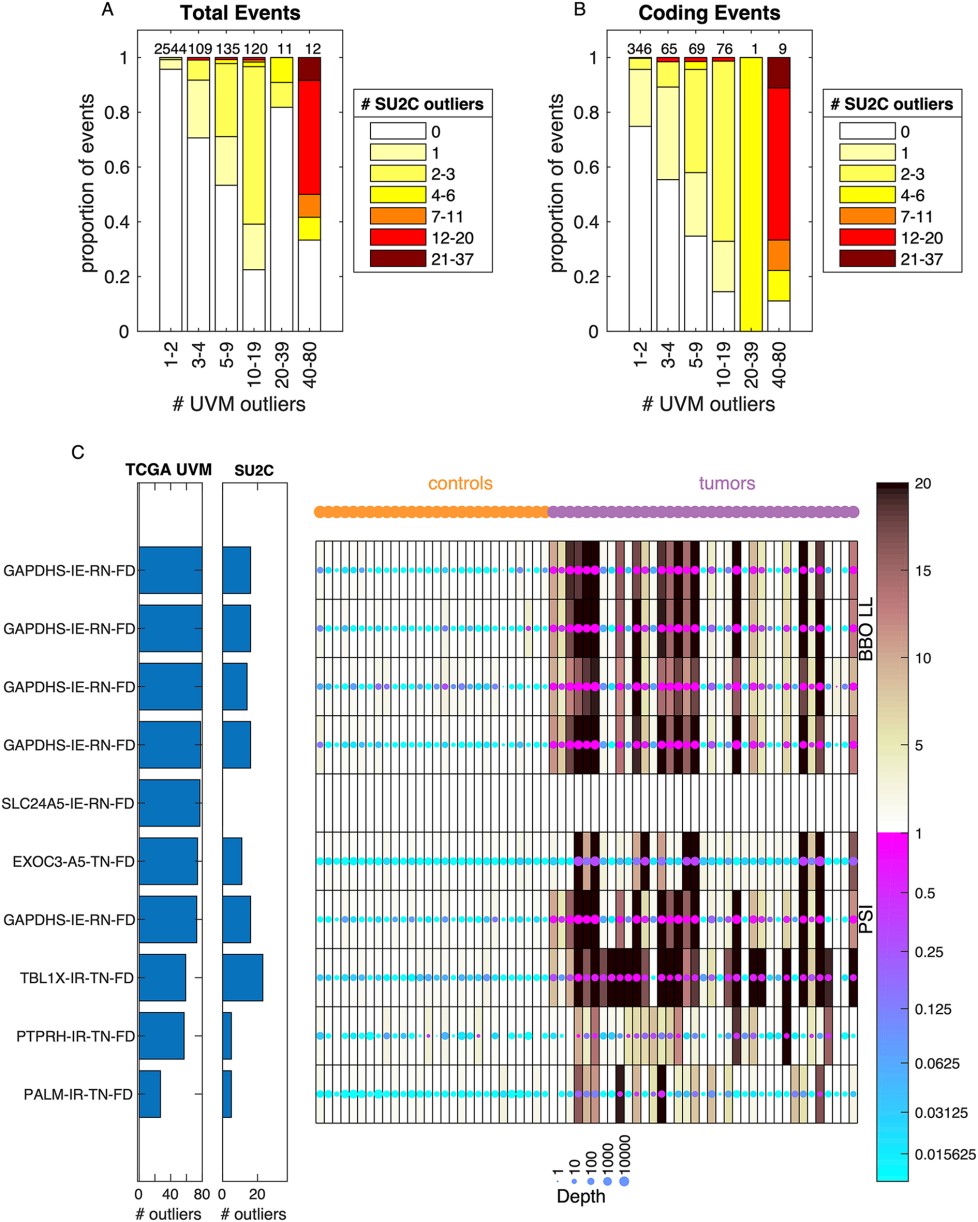
Common melanoma associated splice events are shared between cohorts. (**A**) Comparison of number tumors meeting outlier criteria between cohorts. Along the x-axis, events are binned by the number of tumors meeting the outlier threshold in the TCGA cohort. The total number of events in each bin is indicated at the top of each bar. Within each bar, the events are binned by the total number of tumors meeting the outlier threshold in the SU2C cohort, and the proportion of events in each bin is indicated by the color. (**B**) Same as (**A**) but only including events with predicted protein coding changes. (**C**) Heatmap of the data in the SU2C cohort for the 10 events with predicted coding sequence changes that are found in more than 20 tumors in the TCGA cohort. Each row is an event and each column is a sample, with the controls on the left and the tumors on the right. The color of each dot represents the PSI and the size of each dot represents the coverage at the event. The shading behind the dot indicates the Bisbee outlier score. The bar graphs to the left of the heatmap indicate the number of tumors meeting the outlier threshold in each cohort. The labels on the left indicate the gene name, event type (IE—intron exclusion, A5—alternative 5′, IR—intron retention), and the effect type (RN-FD—frame disruption in the control samples predicted to result in novel sequences, TN-FD frame disruption in the tumor samples predicted to result in novel sequences).

In addition to observing splice events associated with SF3B1 mutation, we also observed splice events common across the TCGA uveal melanoma cohort, irrespective of SF3B1 mutation status. In order to validate this finding, we performed the Bisbee splicing analysis on an independent melanoma cohort, consisting of 37 patients with BRAF wild-type recurrent tumors including 13 cutaneous, 7 mucosal, 10 uveal, 5 acral, 1 melanoma of unknown primary. We performed the Bisbee outlier analysis using both the GTEx excluding testis as the reference and a set of 28 normal tissue or cell lines sequenced at the same institution as the reference and took the minimum score of the two analyses. We compared the number of patients passing the outlier threshold for each event between the two datasets. We identified 23 splice events with 20 or more tumors meeting the outlier criteria in the TCGA dataset, and found that 10 of these events were also detected as outliers in at least one of the SU2C tumors (Fig. 6A). When only considering events with predicted protein sequence changes, there are ten events meeting the outlier criteria in 20 or more of the TCGA tumors and nine of these events are detected as outliers in at least one of the SU2C tumors (Fig. 6B). These nine events identified in both datasets include five intron exclusion events in GAPDHS predicted to result in novel sequence in the reference samples. There is also an alternative 5 prime site in EXOC3, and intron retention in TBL1X, PTPRH, and PALM that are predicted to result in novel sequence in the tumors (Fig. 6C). The intron retention event in SLC24A5 was not detected by SplAdder in the SU2C dataset.

## Discussion

We have developed a new package for splicing data analysis called Bisbee. Bisbee provides functions for differential splicing analysis, splicing outlier analysis, and protein effect prediction. Using a dataset with matched RNAseq and mass spectrometry data on normal human tissues we constructed a truth set to benchmark differential splicing and outlier methods, including Bisbee. We found that Bisbee’s differential splicing approach had substantially better enrichment of proteomics-confirmed events than the other approaches. Bisbee’s outlier test also outperformed other outlier approaches. We demonstrated the utility of the approach in both a rare disease and a cancer context.

The Bisbee package goes beyond many other RNAseq-focused splicing analysis packages by generating protein sequences for the observed splice events. While there are other tools for transcriptomic-proteomic integration, they are generally focus on generating comprehensive databases for mass spectra matching rather than on generating high confidence protein sequence predictions from the RNAseq data. By using Bisbee to generate a database of patient specific protein isoforms from RNAseq data, and then using mass spectrometry to detect which ones have protein level evidence, one could identify high confidence disease-specific protein isoforms for further characterization. The protein domains impacted and other downstream functional predictions from the protein sequences enable further insight into the impact of splicing alterations and can identify splicing-derived pathogenic variants that would go undetected by DNA sequencing alone.

The Bisbee pipeline currently relies on SplAdder for splice event detection^14^. While the work presented here as well as previous work demonstrate that SplAdder is a robust tool for splice event detection, in the future, we plan to adapt Bisbee to work with input from other splice detection tools and benchmark against SplAdder. Bisbee is also limited to the types of splice events detected by SplAdder. Other event types that are not currently detected include alternate first exon, alternate terminal exon, and complex events involving more than one type of alteration. Another limitation of the current approach is that it relies on short read sequencing and does not attempt to assemble a full-length transcript but rather focuses on the local changes in the transcript and protein sequences. An expansion of the approach to incorporate long read data would be useful for enabling full length sequence analysis. Currently, Bisbee only offers two statistical tests: comparison between two groups and outlier detection compared to a reference set. Future work may extend the methods to test for associations with continuous variables or other more complex experimental designs.

The differential splicing test in Bisbee uses a novel beta binomial model to test for differences in PSI. Most differential splicing tools, including the SplAdder test included in our evaluation, test for differences in expression level of the splice isoform, controlling for the overall expression level of the gene. Many of the events that are highly significant in SplAdder’s test have relatively small differences in mean PSI between the two groups (Fig. 4B). In order to identify events with more substantial differences in mean PSI between the two groups, one may directly test for a difference in PSI values using a t-test. However, we have shown that the beta binomial model implemented in Bisbee better addresses the relationship between PSI measurement accuracy and depth. Bisbee is able to detect both low coverage events with dramatic differences in PSI and high coverage events with small differences in PSI (Fig. 4A).

Bisbee is the second splicing tool that we are aware of to offer an outlier detection test. This test is intended for identifying splice isoforms unique to an individual patient compared to a set of reference samples. We were not able to compare directly to the other splice outlier detection tool (leafCutterMD) as it does not report splice events in a way that is amenable to protein sequence generation. The case studies we presented illustrate the utility of the outlier approach in both the rare disease and cancer research.

Currently, the collective use of whole-exome sequencing (WES), overlayed with RNA-Seq data, has enhanced the identification of disease-causing splice mutations and has significantly improved the diagnostic rate of rare diseases. We provided a case study of a rare disease whereby three patients with known pathogenic splice variants were available. We performed differential splicing analysis and the likely pathogenic or causal event was the highest scoring of those predicted to generate a novel amino acid sequence, illustrating how the protein level annotation can aid variant prioritization. Outlier analysis is an important approach in studying rare disease as often more than one case is not available. These cases were difficult to detect by the outlier analysis alone due to the very low coverage at the event locus. However, it is conceivable that the Bisbee output could still help identify the causal variant when examined alongside with candidate variants from WES and knowledge of the phenotype and underlying pathways is exploited.

Previous work has suggested that splicing dysregulation in cancer may be a greater source of tumor specific antigens than somatic point mutations^3, 25^. Application of the Bisbee outlier test to cancer patient samples may enable the discovery of tumor-specific splicing-derived neoantigens, which could be therapeutic or vaccine targets. Splice events that are both outliers compared to normal tissues and differentially spliced between SF3B1 mutant and wild-type tumors are promising candidates as tumor-specific neoantigens, as many of these are predicted to generate novel sequences through frame disruptions and insertions in the tumor-specific isoforms (Fig. 5C). SF3B1 mutant uveal melanomas have better prognosis than SF3B1 wild-type^50^. We hypothesize that the tumor-specific splice isoforms associated with SF3B1 mutations may act as antigens enabling better immune control of the tumors. The protein sequence output from Bisbee would facilitate in silico MHC binding prediction to further investigate the potential immunogenicity of these splice variant generated neoantigens.

We also detected splice outliers common to uveal melanoma regardless of SF3B1 mutation status, and these results showed strong concordance in an independent melanoma cohort. Interestingly, events with predicted protein sequence impact showed stronger concordance than those with no predicted impact (Fig. 6A, B). Nine of the ten events identified as common splice variant outliers with protein impact in the TCGA uveal melanoma dataset were also detected in the SU2C melanoma dataset. These melanoma associated splice variants included several intron retention events in GAPDHS, with the tumors having lower expression of the intron-retained transcripts compared to the normal reference tissues. GAPDHS is typically expressed in sperm, but not in normal somatic tissues, and has previously been shown to be expressed in melanoma^51^. We hypothesize that we are seeing these events in GAPDHS due to expression of the immature transcript in the normal tissues. Four melanoma associated splice events were identified that were predicted to lead to frame disruptions in the tumors, resulting in novel protein sequence. These events are most promising for further investigation as candidate targets in melanoma.

In summary, the Bisbee package is able to predict protein sequences of both known and novel protein isoforms. It provides a more statistically powerful differential splicing test than existing methods. It also provides an outlier detection approach, which will be useful in a number of different contexts, including cancer and rare disease. The Bisbee package is publicly available, and should enable the robust detection of aberrant splicing.

## Methods

### Description of datasets used

For initial evaluation and optimization of the differential and outlier splicing test implemented in Bisbee, we compared the distribution of the likelihood ratios between tests involving samples from the same tissues compared to samples from different tissues. For this analysis GTEx SplAdder results were downloaded from GDC (https://gdc.cancer.gov/about-data/publications/PanCanAtlas-Splicing-2018)^3^. For the differential splicing evaluation, 50 random pairs of tissues were selected, six random samples were selected from each tissue, and 100,000 events were selected for each tissue pair. The beta binomial differential splicing test was applied to grouping the samples into two groups of three replicates within each tissue as well as between the pairs of different tissues. For the outlier evaluation, 12 tissues with at least 100 samples were selected and 80 samples were randomly selected for fitting the model and 20 were selected for determining the outlier scores.

For further evaluation and benchmarking, we identified a dataset where RNAseq and mass spectrometry data were available on the same set of tissues^8^. We selected a total of seven tissues including four lower GI tissues (colon, duodenum, rectum, and small intestine) to serve as the reference set for relevant outlier analyses and three diverse tissues for comparison purposes (brain, ovary, and tonsil). RNAseq reads were downloaded from ArrayExpress (E-MTAB-2836) and aligned to the human reference genome (GRCh38) using star 2.7.3a two pass basic mapping mode and splice events were detected using SplAdder v2.3.0 with default parameters. Two databases for searching were constructed in order to separately assess the FDR for known and novel sequences. The first consisted of Bisbee prot known splice isoforms as well as canonical sequences from Ensembl, and the second included only novel sequences. The LC–MS/MS spectra were downloaded for 7 tissue types from the EBI PRIDE database (PXD010154). The spectra were searched using Mascot (Matrix Science, London, UK; version 2.6.0) through Proteome Discoverer 2.4 (Thermo Fisher Scientific, Waltham, MA), allowing for oxidation (Met) and carbamidomethylation (Cys) dynamic and static modifications, respectively. A maximum of two missed cleavages were allowed with fragment mass tolerance of 0.02 Da and precursor mass tolerance of 10 ppm. FDR thresholds for PSMs, peptides and proteins were set at 0.01, with a minimum of 1 peptide required for protein identification. Peptides that mapped to protein products of more than one gene were excluded from downstream analysis. Peptides that matched exclusively to only one protein isoform sequence were taken as evidence for that isoform. Events where only one isoform was detected in one tissue and the other isoform detected in a different tissue were taken as protein-level evidence of tissue-specific splicing.

For an example use case, three Leigh syndrome and five unaffected control fibroblast cell lines from the study participants were established. RNA was extracted and sequenced by Illumina paired end sequencing and aligned to the reference genome using STAR. Please see the supplementary methods for details of the cell culture, sequencing, and alignment.

For the uveal melanoma analysis, TCGA SplAdder results were downloaded from GDC (https://gdc.cancer.gov/about-data/publications/PanCanAtlas-Splicing-2018)^3^. The SF3B1 mutation status was obtained from cBioportal (https://bit.ly/3hagZvp)^52^.

We used an independent set of melanoma patients for comparison with the TCGA melanoma dataset, referred to here as the SU2C melanoma cohort. RNA was extracted from core needle biopsies, sequenced using Illumina paired end sequencing, and reads were aligned to the reference genome using STAR. Please see the supplementary methods for additional details on the cohort, sequencing, and alignment.

### Splice event protein sequence prediction

In order to generate protein sequences corresponding to each splice event, we use known transcript sequences from Ensembl as a starting point. We first determined whether each isoform of the event exists with any known transcripts, by comparing the event junction coordinates to the exon coordinates (retrieved using the python package pyEnsembl) of protein coding transcripts for that gene. Each transcript is categorized as matching isoform one, isoform two, or neither for the splice event. For each transcript matching the isoform one, the isoform one junctions are removed and replaced with the isoform two junctions to make the altered sequence, and vice versa for those matching isoform two. The region of altered amino acids is found by aligning the two sequences. If the altered amino acid sequence is not found in any of the canonical sequences, the event is categorized as novel. If no transcript is found that matches either isoform, no sequence is generated and the event’s effect is categorized as unknown. In order to narrow down to one pair of protein sequences per event, the sequences are prioritized as follows: (1) pair of known transcripts, (2) longest altered amino acid sequence, (3) longest starting isoform sequence.

### Differential splicing test (Bisbee diff)

Read counts for a splice variant are modeled as following a beta binomial distribution. Here the number of reads supporting the first isoform is the number of successes, the total number of reads covering the event is the number of trials, and the expected PSI (percent spliced in) value across the samples of interest is represented by the beta distribution. The beta distribution is reparameterized as $\psi =\frac{\alpha }{\alpha +\beta }$ and $\omega =\alpha +\beta$. $\psi$ represents the expected value of the beta distribution and $\omega$ affects the sharpness of the distribution, but is more intuitive then actual variance of the beta function, which is a much more complex function of $\alpha$ and $\beta$. In the one group model, all of the samples are assumed to have the same underlying distribution of PSI values and a maximum likelihood estimate is made for $\psi$ and $\omega$. In the models below, ${I}_{n}$ indicates the number of reads supporting the first isoform for sample n, ${T}_{n}$ indicates the total number of reads supporting either isoform for sample n, and ${N}_{A}$ indicates the total number of samples.

$$L_{A} = \mathop \sum \limits_{n = 1}^{{N_{A} }} betabinomial_{pdf} \left( {I_{n} ,T_{n} ,\omega_{A} \times \Psi_{A} ,\omega_{A} \times (1 - \Psi_{A} )} \right)$$

In the two-group model, it is assumed that the two groups have different expected PSI values, but similar distribution shapes, so the two groups have different values of $\psi$ but the same $\omega$.

$$\begin{aligned} L_{G} & = \mathop \sum \limits_{n = 1}^{{N_{1} }} betabinomial_{pdf} \left( {I_{n} ,T_{n} ,\omega_{G} \times \Psi_{1} ,\omega_{G} \times \left( {1 - \Psi_{1} } \right)} \right) \\ & \quad + \mathop \sum \limits_{n = 1}^{{N_{2} }} betabinomial_{pdf} \left( {I_{n} ,T_{n} ,\omega_{G} \times \Psi_{2} ,\omega_{G} \times \left( {1 - \Psi_{2} } \right)} \right) \\ \end{aligned}$$

For both the one group and two group models we find the values of the parameters that maximize the sum of the probability densities across the data points. In fitting the model, we use logistic transformations as shown below to constrain $\omega$ to be greater than 2 and less than ${\omega }_{M}$ and constrain $\Psi$ to be between 0 and 1. $a$ and $b$ are the parameters that are optimized in order to ensure that the values of $\Psi$ and $\omega$ remain within the constraints.

$$\omega = \frac{{\omega_{M} }}{{1 + e^{a} }} + 2, \Psi = \frac{1}{{1 + e^{b} }}$$

The difference in the sum of the log probability densities across the two models is used to identify that events have different underlying PSI distributions in the two groups.

$$LR = log\left( {L_{A} } \right) - log\left( {L_{G} } \right)$$

### Splicing outlier test (Bisbee outlier)

As in the two-group test, the read counts are assumed to follow a beta binomial distribution. The beta distribution parameters are found that maximizes the sum of the log probability densities across a set of reference samples.

$$\{{\alpha }_{R},{\beta }_{R}\} =argmax\left({\sum }_{n=1}^{{N}_{R}}{betabinomial}_{pdf}\left({I}_{n},{T}_{n},{\alpha }_{R},{\beta }_{R}\right)\right)$$

Here ${I}_{n}$ indicates the read count supporting the isoform with mean PSI < 0.5 across the samples. Nelder-Mead optimization^53^ (or BFGS if Nelder-Mead fails) is used to find the maximum likelihood values of ${\alpha }_{R},{\beta }_{R}$. The reparameterizations below are used to constrain ${\alpha }_{R}$ to be between $\frac{1}{{\beta }_{M}}$ and 1 and ${\beta }_{R}$ to be between 1 and ${\beta }_{M}$ so that the beta distribution is strictly decreasing. The values the $a$ and $b$ parameters are optimized to find the maximum likelihood values of ${\alpha }_{R},{\beta }_{R}$ within the constraints.

$$\alpha_{R} = \frac{{1 - \frac{1}{{\beta_{M} }}}}{{1 + e^{a} }} + \frac{1}{{\beta_{M} }},\beta_{R} = \frac{{\beta_{M} - 1}}{{1 + e^{b} }} + 1$$

If zero reads are detected supporting the minor isoform in the reference sample set, alpha is set to one and beta is set as shown below.

$$\beta_{R} = min\left( {\beta_{M} ,\mathop \sum \limits_{n = 1}^{{N_{R} }} T_{N} } \right)$$

For each sample of interest, the log cumulative probability of the major isoform read counts being less than or equal to those observed given the total read depth and the beta distribution fit to the reference sample is used as the outlier score.

$$LL_{s} = log\left( {betabinomial_{cdf} \left( {I_{s} ,T_{s} ,T_{n} ,\alpha_{R} ,\beta_{R} } \right)} \right)$$

### Implementation of other differential splicing methods

For benchmarking we selected SplAdder’s differential splicing test using default parameters as a representative of the generalized linear model approach. We also wanted to include a simple method directly testing differences in PSI values. While a non-parametric test would be more appropriate, as PSI values are unlikely to be normally distributed, we would not have any power to detect differences with only three or four replicates per group. Instead, used a two-sample t-test, with the more conservative assumption of unequal variance, on the PSI values. SplAdder only reports PSI values for samples with a coverage of 10 for a given event, though PSI values can still be calculated from the isoform one and two coverages. We applied the t-test both to all PSI values as well as treating the data points with depth less than 10 as missing data.

### Implementation of other splicing outlier detection methods

We implemented two simple methods using the distribution of PSIs for comparison. The first finds the median absolute deviation from the set of reference samples. Below, ${x}_{s}$ indicates the PSI of the sample s and ${x}_{R}$ indicates the array of PSI values associated with reference samples.

$${MAD}_{s}=\frac{{x}_{s}-median({x}_{R})}{max(median(\left|{x}_{r}-median({x}_{R})\right|),0.01)}$$

The second normalizes to the interquartile range.

$${x}_{s}<{Q}_{25}:{IQR}_{s}= \frac{{Q}_{25}-{x}_{s}}{max({Q}_{75}-{Q}_{25}),0.01)}$$

$${{Q}_{25}\ge x}_{s}\ge {Q}_{75}: {IQR}_{s}=0$$

$${x}_{s}>{Q}_{75}:{IQR}_{s}= \frac{{{x}_{s}-Q}_{75}}{max({Q}_{7t}-{Q}_{25}),0.01)}$$

We applied both of these methods either using all of the PSI values, as well as only using data points with depth less than 10 as missing data.

### Ethics approval and consent to participate

The study protocol and written informed consent for the use of human fibroblast cells of the MFTMT cases and controls was approved by the Western Institutional Review Board (WIRB; study number 20120789). The SU2C melanoma biopsies were collected through a clinical trial (NCT02094872). Ethics review boards at all participating institutions approved the study, which was conducted in accordance with the Declaration of Helsinki and Good Clinical Practice guidelines. All patients provided written informed consent.

## Supplementary Information

Supplementary Information.

## Data Availability

The RNA sequencing data of the MTFMT cases and controls will be deposited in dbGap. The SU2C RNA sequencing data is available in dbGap under accession phs001786.v1.p1. The Bisbee package is available at https://github.com/tgen/bisbee.
