# Supplementary material for: Improved methods for RNAseq-based alternative splicing analysis
Source: Sci Rep. 2021 May 24;11:10740. doi: 10.1038/s41598-021-89938-2 (PMC8144374; doi:10.1038/s41598-021-89938-2)
Supplement: Supplementary file 1 — Supplementary Information. [file 41598_2021_89938_MOESM1_ESM.pdf]

Bisbee: A proteomics validated analysis package for detecting differential splicing, identifying splice outliers, and predicting splice event protein effects

Authors: Rebecca F. Halperin[1**], Apurva Hegde[2], Jessica D. Lang[3], Elizabeth A. Raupach[3], C4RCD Research Group, Christophe Legendre[3], Winnie S. Liang[3,4], Patricia M. LoRusso[5], Aleksandar Sekulic[6], Jeffrey A. Sosman[7], Jeffrey M. Trent[3], Sampathkumar Rangasamy[4], Patrick Pirrotte[2*], Nicholas J. Schork[1*]

[1]Quantitative Medicine and Systems Biology Division, [2]Collaborative Center for Translational Mass Spectrometry, [3]Integrated Cancer Genomics Division, [4]Neurogenomics Division, Translational Genomics Research Institute, Phoenix AZ, [5]Yale Cancer Center, New Haven CT, [6]Mayo Clinic, Scottsdale AZ, [7]Northwestern Medicine, Chicago IL
* Co-senior authors
**Corresponding author

Author emails: RFH - rhalperin@tgen.org, AH - ahegde@tgen.org, JDL - jlang@tgen.org, EAR - eraupach@tgen.org, CL - clegendre@tgen.org, WSL - wliang@tgen.org, PML - patricia.lorusso@yale.edu, AS - sekulic.aleksandar@mayo.edu, JAS - Jeffrey.Sosman@nm.org, JMT - jtrent@tgen.org, SR - srangasamy@tgen.org, PP - ppirrotte@tgen.org, NJS - nschork@tgen.org

Supplemental Methods

Description of example datasets

The Leigh syndrome and control study participant cell lines were established from 3 mm skin biopsy punches and cultured for 2 weeks in primary fibroblast media containing the following: Minimal Essential Media (Invitrogen, Carlsbad, CA, USA), 20% FBS (American Type Culture Collection, Manassas, VA, USA), Penicillin/Streptomycin and Amphotericin (Sigma-Aldrich, St. Louis, MO, USA), and Plasmocin (InvivoGen, San Diego, CA, USA) (Villegas & McPhaul 2005). RNA was extracted from the study fibroblast using the total RNA Purification Kit (Norgen Biotek Corp, On, Canada) , and RNA Library Preparation was done with the KAPA mRNA HyperPrep Kit with RiboErase (Kapa Biosystems) according to the manufacturer's protocol. Barcoded libraries were pooled (8-plex) and run on two lanes of a flow cell on an Illumina NovaSeq 6000 System. Sequencing was performed using 2 x 100 bp reads. The dataset was aligned to the human reference genome GRCh37 using STAR (v2.4.0) [52], and quality control was performed using Picard RnaSeqMetrics (v1.128).

For the SU2C melanoma cohort, biopsies were collected as part of a clinical trial (NCT02094872), where inclusion criteria included patients aged ≥18 years with metastatic or

locally advanced and unresectable *BRAF*wt melanoma who had progressed following previous treatment. RNA was extracted from frozen core needle biopsies using the Qiagen AllPrep Kit. Illumina's TruSeq RNA Sample Preparation V2 Kit was used to construct libraries which were sequenced on the Illumina HiSeq2500 for 2x100 reads. RNA FASTQs were aligned to the human reference genome GRCh37using STAR 2.3.1z [52].

Supplemental Figure 1. Mass spectrometry peptide mapping

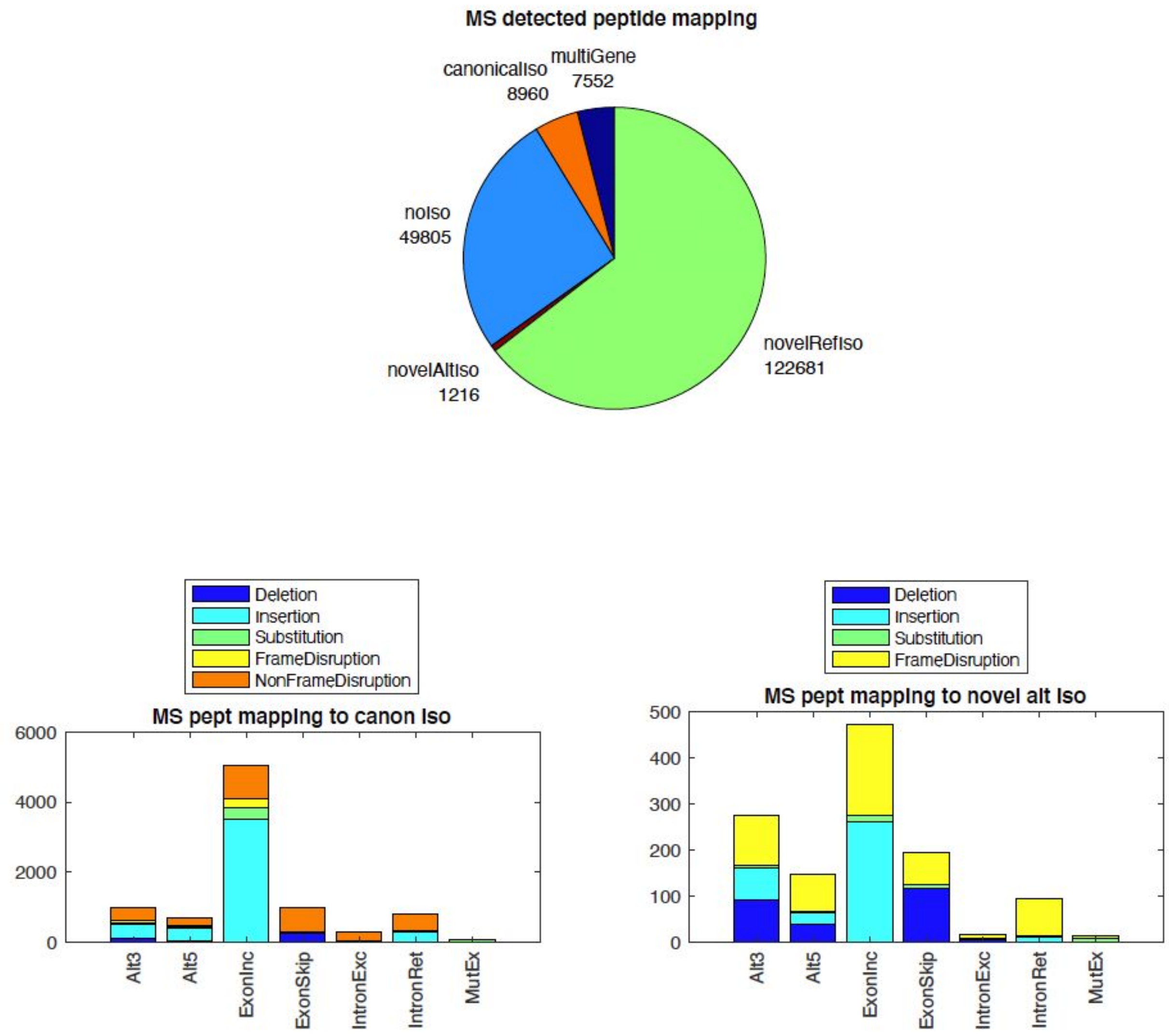

Pie chart illustrates the breakdown of peptides detected by mass spectrometry mapping to splice isoforms. "multiGene" indicates peptides mapping to more than one gene which were excluded from further analysis. "canonicalIso" indicates peptides that map to an isoform of a canonical spice event, "noIso" indicates peptides that map to a part of a protein not involved in an alternative splice event, "novelAltIso" indicates peptides that map to a the alternative (novel) isoform of a novel splice event, and "novelRefIso" indicates peptides that map to the reference isoform of a novel spice event. The barcharts show the breakdown of the peptides mapping to

canonical isoforms (left) or the novel isoforms (right) by splice event type and protein level effect. "NonFrameDisruption" indicates that the splice event was predicted to lead to a frame disruption and the peptide mapped to the in frame isoform.

Supplemental Figure 2. Length of altered amino acid sequence generated by splice events

Boxplots illustrate distribution of the length of amino acid sequence differing between the two isoforms for splice events on a log scale. Red bars show the distribution for all of the predicted events and blue bars show the distribution only for events with confirmed protein expression for each event type. As expected, events generating longer altered regions are more likely to be

detected in mass spectrometry data. Events involving wild-type isoforms tend to generate longer affected regions than those generating novel isoforms.

Supplemental Figure 2. Parameter optimization.

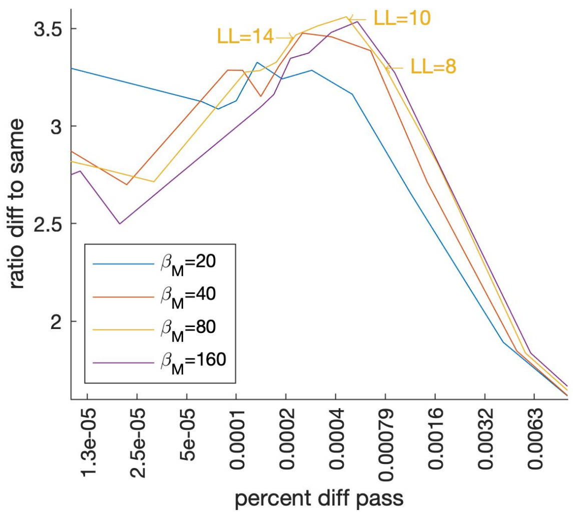

a) *The differential beta binomial test was run on random sets of samples from the same tissue or different tissues selected from GTEx with different values of the* ω_M *parameter. The ratio of percentage of events that are higher than a given LR threshold in the different tissue comparison compared to the same tissue comparison is plotted against the percentage of events in the different comparison that pass the threshold. b) The outlier model was trained on 12 different tissues with 80 samples in each set using different values of the maxW parameter. Outlier scores were found for each of the 12 models for a different set of test samples selected from the same tissues. The ratio of the percent of data points passing an outlier score threshold between models fit on the same tissue vs different tissues is compared to the percentage of data points from fitting unmatched tissue models that pass the threshold.*

Supplemental Figure 3. Bisbee detects known Leigh syndrome pathogenic splice variants in the MTFMT gene.

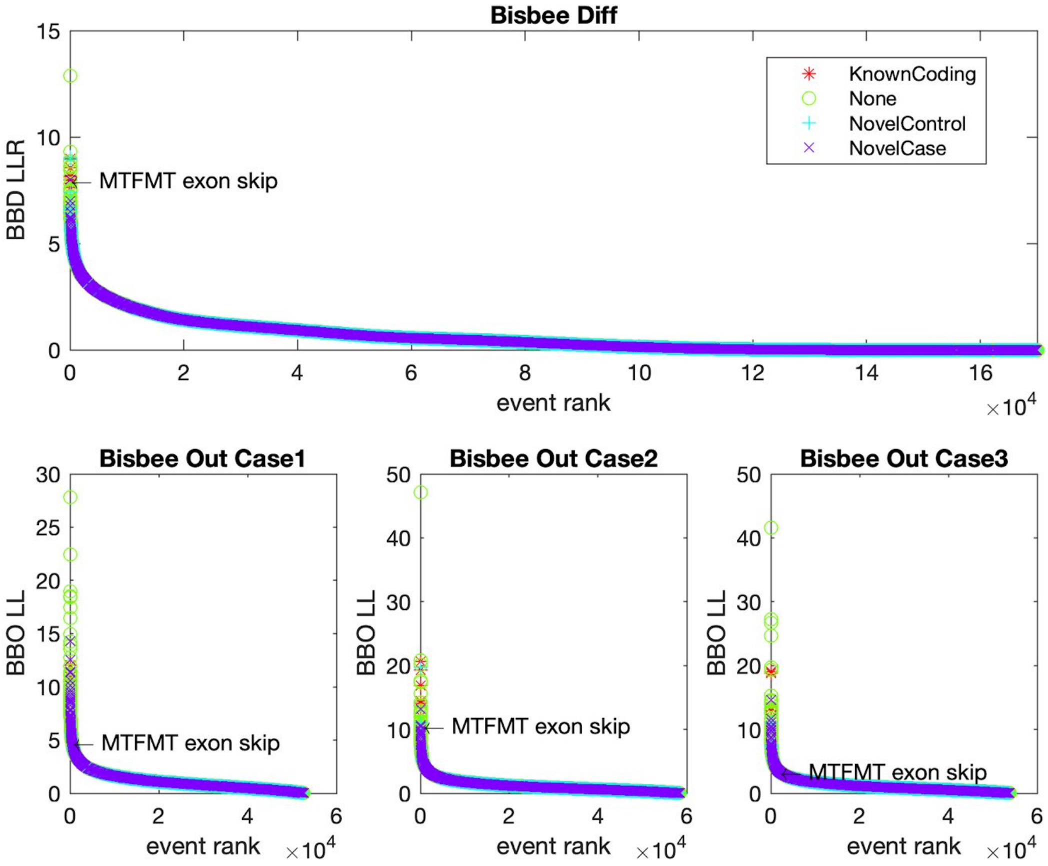

The Bisbee scores are plotted on the y-axis against the rank of the scores on the x-axis in the differential splicing analysis (top) and the outlier analysis for the three cases (bottom). Each point represents a splice event and the color and symbol indicate whether the event is predicted to generate known coding isoforms (red asterisk), none coding or effect not predicted (green circle), novel coding sequence with the novel isoform more highly expressed in the controls (cyan plus) or more highly expressed in the cases (purple x).

Supplemental Table 1. Differential splicing benchmarking summary

Name	1st threshold	Confirmed pass 1st	Total pass 1st	2nd threshold	Confirmed pass 2nd	Total pass 2nd
**Bisbee Diff**	lr>8	32	1760	lr>12	7	196
**SplAdder Test**	p<0.01	3	8870	p_adj<0.05	2	6046
**T-test all**	p<0.01	31	8645	fdr<0.05	1	127

**T-test D10**	p<0.01	30	6448	fdr<0.05	1	103

Summary of protein confirmed events compared to total events at two different thresholds for the four differential splicing methods.

Supplemental Table 2. Outlier test benchmarking

Name	1st threshold	Confirmed pass 1st	Total pass 1st	2nd threshold	Confirmed pass 2nd	Total pass 2nd
**Bisbee Out**	10	43	2947	14	34	1674
**MAD**	20	36	19371	40	30	10859
**MAD-D10**	20	38	3883	25	32	2712
**IQR**	10	40	14146	20	25	8178
**IQR-D10**	8	42	7694	10	40	5597

Summary of protein confirmed events compared to total events at two different thresholds for the five outlier splicing methods.
